# Antibacterial, Antifungal, Cytotoxic, Phytotoxic, Insecticidal, and Enzyme Inhibitory Activities of *Geranium wallichianum*

**DOI:** 10.1155/2012/305906

**Published:** 2012-09-20

**Authors:** Muhammad Ismail, Javid Hussain, Arif-ullah Khan, Abdul Latif Khan, Liaqat Ali, Farman-ullah Khan, Amir Zada Khan, Uzma Niaz, In-Jung Lee

**Affiliations:** ^1^Department of Pharmacy, University of Peshawar, Peshawar 25000, Pakistan; ^2^Department of Biological Sciences and Chemistry, College of Arts and Sciences, University of Nizwa, Nizwa 616, Oman; ^3^Department of Chemistry, Kohat University of Science and Technology, Kohat 26000, Pakistan; ^4^Department of Pharmacy, Kohat University of Science and Technology, Kohat 26000, Pakistan; ^5^Departmen of Chemistry, University of Science and Technology, Bannu 28000, Pakistan; ^6^College of Pharmacy, University of Punjab, Lahore 54000, Pakistan; ^7^School of Applied Biosciences, College of Agriculture and Life Sciences, Kyungpook National University, Daegu 702-701, Republic of Korea

## Abstract

The present study describes the phytochemical investigations of the crude extracts of rhizomes and leaves of *Geranium wallichianum*. The crude extracts were fractionated to obtain *n*-hexane, ethyl acetate, and *n*-butanol fractions, which were subjected to different biological activities and enzyme inhibition assays to explore the therapeutic potential of this medicinally important herb. The results indicated that the crude extracts and different fractions of rhizomes and leaves showed varied degree of antimicrobial activities and enzyme inhibitions in different assays. Overall, the rhizome extract and its different fractions showed comparatively better activities in various assays. Furthermore, the purified constituents from the repeated chromatographic separations were also subjected to enzyme inhibition studies against three different enzymes. The results of these studies showed that lipoxygenase enzyme was significantly inhibited as compared to urease. In case of chemical constituents, the sterols (**2**–**4**) showed no inhibition, while ursolic acid (**1**) and benzoic ester (**6**) showed significant inhibition of urease enzymes.

## 1. Introduction

*Geranium wallichianum* D. Don belongs to family Geraniaceae, commonly called as “Shephred's needles” and locally as “Ratanjot” [1]. It is a perennial herb with thick rootstock, and hairy and robust stem of about 1–4 ft. length, while the blue-colored flowers ranges from 3–8.5 cm in diameter [2]. The herb evidently possesses astringent properties of the genus to a marked degree. The root stock is used in eye troubles and also in the treatment of toothache [3]. The roots of the plant are used in mouth ulceration, dysentery, diarrhea, passive hemorrhage, and leucorrhoea [4]. 

The development of antimicrobial resistance in many pathogenic microbes possesses one of the most serious problems in the control of infectious diseases. Ethanobotanical data have proved useful in the search for new antimicrobial agents and many of these compounds have been isolated directly from medicinal plants [5]. Freedom from insect infestation and contamination is another important consideration in storage of grain and to maintain a high-quality product [6]. Nearly one thousand species of insects have been associated with stored products throughout the world, of which the majority belong to *Coleoptera* (60%) and *Lepidoptera* (8-9%). Pesticides, including residual grain protectants and fumigants are used extensively in grain industry, but the major pest species have developed resistance to most of these materials [7]. This relentless development of resistance is a serious threat to the future use of these materials and consequently, there is an urgent need to develop economically safer and sounder pest control techniques.

Urease is an enzyme responsible for an organism to use urea as nitrogen source to convert it into ammonia and carbon dioxide [8, 9]. It acts as defense protein in plants in systemic nitrogen transport pathways [10], and also plays an important role in the pathogenesis of gastric and peptic ulcer, apart from cancer as well [11]. Urease is directly involved in the formation of infection stones and contributes to the pathogenesis of urolithiasis, pyelonephritis, ammonia, and hepatic encephalopathy, hepatic coma, and urinary catheter encrustation [12]. In agriculture, by contrast, a hydrolysis of fertilizer urea by soil urease, if too rapid, results in an unproductive volatilization of nitrogen and may cause ammonia toxicity or alkaline-induced plant damage. Ureases have also a role in the inactivation of complement, which is a component of host defense mechanism [13]. Due to these diverse functions of this enzyme, its inhibition by potent and specific compounds could provide an invaluable addition for the treatment of infections caused by Urease-producing bacteria [14].

**Figure 1:**
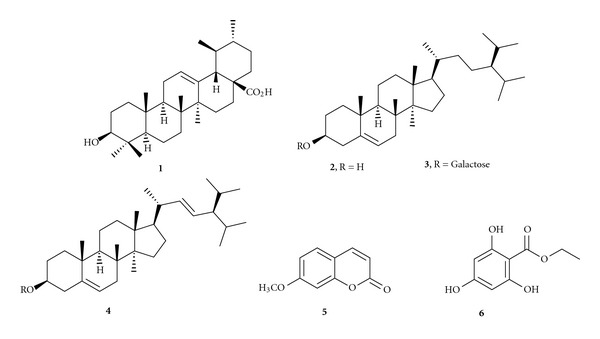
Structures of compounds **1–6**.

In the present investigation, we studied the *Geranium wallichianum* for variety of biological activities including antimicrobial, insecticidal, cytotoxicity, phytotoxicity, and enzyme inhibition studies. Apart from the crude extracts and their resultant fractions, some of the pure compounds (Figure 1) isolated from *Geranium wallichianum* were also evaluated.

## 2. Material and Methods

### 2.1. General

Optical rotations were measured on a JASCO DIP 360 polarimeter. MS spectra were recorded on mass spectrometers JEOL JMS HX 110. NMR spectra were recorded on Bruker NMR spectrometers operating at 400 and 500 MHz (100 and 125 MHz for ^13^C). The chemical shifts values are reported in ppm (**δ**) units, and the coupling constants (*J*) are given in Hz. The biological activities were conducted against the standard assays, and the results are reported in comparison with the positive and negative controls (wherever applicable). All chemicals and orthophosphate and ureases enzymes were purchased directly from Sigma St. Loius, MO, USA. Bacterial and fungal strains were collected from the Institute of Pharmaceutical Sciences, Faculty of Pharmacy, University of Karachi, Karachi, Pakistan, and the respective voucher specimen for all samples were deposited in the herbarium of the department. The solvents: *n*-butanol, ethyl acetate, *n*-hexane, chloroform, and methanol were obtained from Merck, Darmstadt, Germany. All chemicals were of analytical reagent grade and were used without further purification.

### 2.2. Plant Material

The plant *Geranium wallichianum* was collected at flowering stage from Abbottabad, Pakistan in July 1999 and was identified by Prof. Jahandar Shah, Department of Botany, Islamia College Peshawar, Pakistan. A voucher specimen has been deposited at the herbarium of the Botany Department, University of Peshawar, Pakistan.

### 2.3. Extraction and Isolation

The powdered rhizomes of the plat (6.0 Kg) were macerated in methanol (12 L), for 15 days and filtered. The procedure was repeated 3 times. All the three filtrates were combined and concentrated under vacuum at 40°C using rotary evaporator. A brownish-black crude extract (130.5 g) was obtained. Similarly the powdered leaves (5 kg) were macerated in methanol (10 L), and a dark-greenish crude extract (162 g) was obtained by using the same procedure. The crude methanolic extract (125 gm) of the rhizome was suspended in distilled water (500 mL) and partitioned with *n*-hexane (3 × 500 mL), chloroform (3 × 500 mL), ethyl acetate (3 × 500 mL), and *n*-butanol (3 × 500 mL), to yield *n*-hexane (10 g), chloroform (22 g), ethyl acetate (32 g), *n*-butanol (27 g), and aqueous (26 g) fractions, respectively. The crude methanolic extract (152 g) of leaves was also suspended in distilled water (500 mL) and partitioned with *n*-hexane (3 × 500 mL), chloroform (3 × 500 mL), ethyl acetate (3 × 500 mL), and *n*-butanol (3 × 500 mL) to yield the *n*-hexane (14 g), chloroform (25 g), ethyl acetate (22 g), *n*-butanol (28 g), and aqueous (56 g) fractions, respectively.

The ethyl acetate fraction (32 g) of the rhizome extract was subjected to column chromatography by using silica gel packed column and eluting with different polarity solvent systems; five fractions (A–E) were obtained. By repeated column chromatography, compound **1** was obtained from fraction A, when eluted with chloroform : *n*-hexane (3 : 7) as colorless needles (30 mg). Similarly, compound **2** (28 mg) and **3** (35.8 mg) were obtained from fraction B at chloroform : *n*-hexane (2 : 8) as colorless solids. The fraction C and D were subjected to silica gel column chromatography to obtain compound **4** (20 mg) and **5** (35 mg) as amorphous solid, while the column eluted with ethyl acetate : *n*-hexane (8 : 3 and 4 : 6, resp.). Finally, compound **6** (55 mg) was obtained from fraction E at chloroform : *n*-hexane (3 : 7). The structures of all these compounds **1–6** are given in Figure 1.

### 2.4. Antibacterial Activity

*Escherichia coli*, *Bacillus subtillis*, *Shigella flexenari*, *Staphylococcus aureus*, *Pseudomonas auriginosa*, and *Solmenella typhi* strains were used in this assay. Using a single sterile pipette, 0.6 mL of the broth culture of the test organism was added to 60 mL of molten agar, which had been cooled to 45°C, mixed well and poured into a sterile petri dish (for the 9 cm petri dish, 0.2 mL of the culture was added to 20 mL of agar). Duplicate plates of each organism were prepared. The agar was allowed to set and harden and the required number of wells was dug in the medium with help of sterile metallic cork borer ensuring proper distribution of the well in the periphery and one in the center. Agar plugs were removed. Stock solutions of the test samples at a concentration of 1 mg/mL were prepared in sterile DMSO and 100 *μ*L and 200 *μ*L of each dilution was added to the respective wells. The control well received only 100 *μ*L and 200 *μ*L DMSO. Imipenin was used as standard drug. The plates were left at room temperature to allow diffusion and then incubated at 37°C for 24 hr. The Diameter of the zones of inhibition was measured to the nearest mm (the well size also being noted)

(1) %Inhibition  =100−linear  growth  in  test  (mm)linear  growth  in  control  (mm)×100.

### 2.5. Antifungal Activity

*Trichophyton longifusus*, *Candida albicans*, *Candida glaberata*, *Microsporum canis*, *Aspergilus flavusi*, and *Fusarium solani* strains were used in the present study. The antifungal activity of the extracts was evaluated by the agar tube dilution method. The samples (24 mg/mL) were dissolved in sterile DMSO, which served as a stock solution. Sabouraud dextrose agar (SDA) was prepared by mixing 32.5 g sabouraud, 4% glucose agar, and 4.0 g of agar-agar in 500 mL distilled water thoroughly with a magnetic stirrer. Then a 4 mL aliquot was dispensed into screw-cap tubes, which were autoclaved at 120°C for 15 min and then cooled to 15°C. The nonsolidified SDA media was mixed with stock solution. (66.6 *μ*L) giving a final concentration of 400 *μ*g of the extract per mL of the SDA. The tubes were then allowed to solidify in slanted position at room temperature and then inoculated with a piece (4 mm diameter) of an inoculum removed from a seven days old culture of fungi to determine nonmycelial growth; an agar surface streak was employed. Other media supplemented with DMSO and reference antifungal drugs served as a negative and positive control, respectively. Inhibition of fungal growth was observed visually after 7 days of incubation at 28 ± 1°C. Humidity (40–50%) was controlled by placing an open pan of water in the incubator.

Growth in the compound amended media was determined by measuring linear growth (mm) and growth inhibition was calculated with reference to the negative control

(2) %Inhibition  =100−linear  growth  in  test  (mm)linear  growth  in  control  (mm)×100.

### 2.6. Brine Shrimp Lethality Bioassay

Brine shrimp (*Artemia salina* larvae) eggs were hatched in a shallow rectangular plastic dish, filled with artificial seawater, which was prepared by mixing a commercial salt mixture (Instant Ocean, Aquarium System, Inc., Mentor, OH, USA) with double-distilled water. An unequal partition was made in the plastic dish with the help of a perforated device. Eggs (50 mg) were sprinkled into larger compartment, which was placed under the dark condition while the smaller compartment was opened to allow ordinary light. After two days, nauplii were collected. A sample of the test fraction was prepared by dissolving 20 mg of each fraction in 2 mL of methanol. From this stock solution, 1000, 100, and 10 *μ*g/mL was transferred to 12 vials; three for each dilution, and three vials were kept as control having 2 mL of methanol only. The solvent was allowed to evaporate overnight. When shrimp larvae were ready, 1 mL of sea water was added to each vial along with 10 shrimps and the volume was adjusted with sea water to 5 mL per vial. After 24 h, the number of surviving shrimps was counted. Data was analyzed by a Finney computer program [15] to determine the LD_50_. Each experiment was replicated thrice.

### 2.7. Bioassay for Phytotoxicity

This test was performed according to the modified protocol of McLaughlin [16]. The test fractions were incorporated with sterilized E-medium at different concentrations that is, 10, 100, and 1000 *μ*g/mL in methanol. Sterilized conical flasks were inoculated with fractions of desired concentrations prepared from the stock solution and allowed to evaporate overnight. Each flask was inoculated with 20 mL of sterilized E-medium and then, ten *Lemna minor*, each containing a rosette of three fronds was placed on media. Other flasks were supplemented with methanol serving as negative control and reference inhibitor, that is, Parquet serving as positive control. Treatment was replicated three times, and the flasks incubated at 30°C in Fisons Fi-Totron 600 H growth cabinet for seven days, 9000 lux intensity, 56 + 10 rh (relative humidity), and 12 hr day length. Growth of *Lemna minor* in fraction-containing flask was determined by counting the number of fronds per dose and growth inhibition was calculated with reference to negative control.

### 2.8. Insecticidal Bioassay

*Tribolium castaneum*, *Rhyzopertha dominica*, and *Callosbruchus analis* were used to determine the insecticidal activity of the crude extracts and their various fractions. Test samples were prepared by dissolving the 200 mg each of crude extract and their fractions in 3 mL volatile solvent, respectively. Filter paper was cut according to the size of petri plate (9 cm) and put in the plate. Each of the samples was loaded over the filter paper with the help of micropipette. The plates were left for 24 hours in order to evaporate the solvent completely. Then 10 healthy and active insects of same size and age were added in each petri plate along with positive control which contains the standard drug, Permethrin and incubated at 27°C for 24 hours with 50% relative humidity in growth chamber. Each of the test samples was a triplicate. The number of survivals for each species was counted on the next day. Percentage mortality in each case was calculated with the help of the following formula:

(3) $$\begin{array}{c} \%\text{Regulation}=100-\frac{\text{No}\;\;\text{of}\;\;\text{insects}\;\;\text{alive}\;\;\text{in}\;\;\text{test}}{\text{No}\;\;\text{of}\;\;\text{insects}\;\;\text{alive}\;\;\text{in}\;\;\text{control}}\times 100. \end{array}$$

### 2.9. Enzyme Inhibition Assay

*In vitro* enzyme inhibition activities of the crude methanolic extract and various fractions of the leaves and underground stem of *Geranium wallichianum* were carried out against lipoxygenase and urease enzymes. Lipoxygenase inhibitory activity was measured by slightly modifying the spectrometric method developed by Tappel [17]. About 160 *μ*L of 0.1 mM sodium phosphate buffer (pH 7.0), 10 *μ*L of the sample solution and 20 *μ*L of lipoxygenase solution were mixed and incubated for 5 min. at 25°C. The reaction was then initiated by the addition of 10 *μ*L linoleic acid solution (substrate), with the formation of (9Z, 11E)-(13S)-13-hydroperoxyoctadeca-9, 11-dienoate; the change of absorbance was followed for 10 minutes. The test sample and the control were dissolved in 50% ethanol. All the reactions were performed in triplicate. The IC_50_ values were then calculated using with EZ-Fit Enzymes Kinetics program. %inhibition = (*E* − *S*)/*E* × 100, where *E* is the activity of the enzyme without test compounds and *S* is the activity of enzyme with test compound. For urease enzyme inhibitory activity, reaction mixtures comprising 25 *μ*L of enzyme (Jack bean urease) solution and 55 *μ*L of buffers containing 100 mM urea were incubated with 5 *μ*L of each of the test compounds, respectively, (1 mM concentration) at 30°C for 15 minutes in 96-well plates. Urease inhibitory activity was determined by measuring ammonia production using the indophenol's method as described [18]. Briefly, 45 *μ*L each of phenol reagent (1% w/v phenol and 0.005% w/v sodium nitroprusside) and 70 *μ*L of alkali reagent (0.5% w/v NaOH and 0.1% active chloride NaOCl) were added to each well. The increasing absorbance at 630 nm was measured after 50 min, using a microplate reader (Molecular Device, USA). All reactions were performed in triplicate in a final volume of 200 *μ*L. The results (change in absorbance per min.) were processed by using Soft Max Pro software (Molecular Device, USA). All the assays were performed at pH 8.2 (0.01 M-K_2_HPO_4_·3H_2_O, 1 mM EDTA, and 0.01 M LiCl). Percentage inhibitions were calculated from the formula, and thiourea was used as the standard inhibitor of urease

(4) $$\begin{array}{c} \%\text{inhibition}=100-\left(\frac{\text{OD}\;\;\text{test}\;\;\text{well}}{\text{OD}\;\;\text{control}}\right)\times 100. \end{array}$$

## 3. Results

Antibacterial actions of *G*.* wallichianum* rhizome and leaves crude extracts as well as their respective resultant fractions were assayed by agar diffusion method. The rhizome crude extract showed antibacterial effect of 57.69, 54.54, and 45.16% against *P*. *aeruginosa*, *S*. *aureus* and *B*. *subtilis*, respectively, (Table 1). Besides, ethyl acetate had 50.01% antibacterial effect against *S*. *aureus*.

The leaves crude extract showed 30.76 and 29.03% inhibitory effect against *P*. *aeruginosa* and *B*. *subtilis*, respectively. All of its fractions were found to be inactive, except ethyl acetate fraction, which exhibited 21.21 and 19.51% activity against *S*. *flexenari* and *S*. *aureus*, respectively (Table 2).

The results of the antifungal activities of extracts and fractions of rhizomes are summarized in Table 3. According to the results, the growth of *F*. *solani* was inhibited 75 and 70% by crude extract and its ethyl acetate fraction, respectively. Crude extract also showed moderate activity against the growth of *C*. *albicans*, and *M*. *canis*. The other fraction did not have any significant effect on the linear growth of various fungi.

The results of cytotoxic activity of the *G*. *wallichianum* rhizome extract along with its resultant fractions using brine shrimp bioassay is shown in Table 4. Ethyl acetate had considerably significant cytotoxicity followed by crude extract while the other fractions showed minimal bioactivity.

The phytotoxicity of the *G*. *wallichianum* rhizome crude extract and fractions was assayed against different concentrations, and the results are summarized in Table 5.

The *G*. *wallichianum* rhizome crude extracts as well as the respective fractions were screened to asses the insecticidal activity by direct contact method. The maximum insecticidal activity of 46.6 and 66.6% were shown against *R*. *dominica* by the chloroform and ethyl acetate fractions, respectively, as shown in Table 6.

The enzyme inhibition studies on the crude extracts of rhizomes and leaves of *G*. *wallichianum* were carried out against lipoxygenase and ureases. The leaves extract showed no activity against any of the tested enzymes, however the rhizome extracts showed varied activities and the results are summarized in Table 7. The enzyme inhibition studies on the pure compounds **1**–**6** are summarized in Table 8. Compounds (**6** and **3**) showed significantly higher enzyme inhibition activity against lipoxygenase as compared to other compounds (**1**, **2**, **4**, and **5**). In case of urease inhibition, compound **1** and **6** showed higher enzyme activity.

## 4. Discussion

In continuation of our studies on the phytochemical investigations of rhizomes and leaves extract of the plant *G*. *wallichianum*, we fractionated the crude extracts into different fractions on the basis of increasing polarity of organic solvents. Previously, we reported [19] the isolation and characterization of compounds **1–6**, along with the antioxidant activities of different fractions. In the present study, the crude extracts of rhizomes and leaves of *G. wallichianum* were subjected to solvent-solvent extraction to get *n*-hexane, chloroform, ethyl acetate, *n*-butanol, and aqueous fractions. These fractions were then subjected to different biological activities to get insight into therapeutic potential of the medicinally important plant. Furthermore, the purified compounds **1–6** were also subjected to various enzyme inhibition studies and those results are also included in the present paper.

The antibacterial activity studies indicated the *B*. *subtilis* being most effective against *P*. *aeruginosa*. All the fractions of rhizome extract showed no antibacterial effect, except ethyl acetate fraction, which showed activity of 50, 38.7, and 30.3% against *S*. *aureus*, *B*. *subtilis*, and *S*. *flexenari*, respectively.

Antifungal action of *G*. *wallichianum* rhizome and leaves crude extracts along with their respective resultant fractions was assayed by tube dilution method. The rhizome crude extract showed antifungal effect of 75, 65, 60, and 55% against *F*. *solani*, *M*. *canis*, *C*. *albicans*, and *C*. *glaberata*, respectively, being most effective against *F*. *solani*. All the fractions of rhizome extract did not exhibit any antifungal effect, except ethyl acetate fraction, which showed activity against *C*. *albicans*, *C*. *glaberata*, *M*. *canis*, and *F*. *solani* by 50, 50, 52, and 70%, respectively.

The ethyl acetate fraction was found to be the most effective against brine shrimps, whereas the lethality affect was 98.33% with LC_50_ value of 333.256, suggesting that this effect is concentrated in the ethyl acetate fraction. The ethyl acetate fraction could thus be considered as the potential fraction, which might be containing some potential cytotoxic compounds.

In phytotoxicity study, *G*. *wallichianum* rhizome crude extract has shown significant effect against *Lemna minor* at all test doses (10–1000 *μ*g/mL), while all its resultant fractions were found less effective, in comparison to the parent extract.

The rhizome crude extract and its aqueous fraction were found to be effective against *Tribolium castaneum* and *Rhyzopertha dominica*, respectively, while ethyl acetate fraction showed effectiveness against all three type of tests insects and the chloroform fraction only against *Tribolium castaneum* and *Rhyzopertha dominica*.

From the results of enzyme inhibition studies, it is found that rhizome crude extract, ethyl acetate, and aqueous fractions have shown enzyme inhibitory activity of 26.9, 47.5, and 45%, respectively, against lipoxygenase, while the inhibitory activity against Jack Bean urease (J.B. urease) and *Bacillus pasteurii urease* (B.P. urease) exhibited by crude extract was 52 and 57%, respectively, whereas for ethyl acetate fractions was 83 and 86%, respectively.

The pure compounds **1–6** isolated and characterized [19] from *G*. *wallichianum* as a result of repeated column chromatography were also subjected to enzyme inhibition assays. Ursolic acid (**1**) caused inhibition of J.B. Urease and B.P. urease by 52 and 57%, respectively, (Table 8). **β**-sitosterol (**2**), **β**-sitosterol-glactoside (**3**), and stigmasterol (**4**) have shown the activity only against lipoxygenase with 37.5, 33, and 40% inhibition, respectively. 2,4,6-trihydroxyethylbenzoate (**6**) was found to be active against all tested enzymes, and the inhibition was 100, 69 and 66% against lipoxygenase, B.P. urease, and J.B. urease, respectively (Table 8). Compound **5** named herniarin was inactive against all three types of enzymes.

## 5. Conclusions

The data presented in the present paper indicated that *G*. *wallichianum* exhibited a variety of biological activities, such as antibacterial, antifungal, cytotoxic, phytotoxic, insecticidal, and enzyme inhibition. The rhizomes of the plant were found to be more effective than the leaves. Thus, the present paper provides an evidence for the various therapeutic uses of the plant in a wide range of disorders and human illness.

## Figures and Tables

**Table 1:** Antibacterial activity of crude extract and fractions of rhizomes (Imipenem was used as standard drug and all the readings were recorded on the basis of zone of inhibition (mm) as compared to standard).

Bacteria	Standard zone of inhibition	Crude extract zone (mm)	*n*-hexane zone (mm)	Chloroform zone (mm)	Ethyl acetate zone (mm)	*n*-butanol zone (mm)	Aqueous zone (mm)
*E. coli*	30	0	0	0	0	0	0
*B. subtillis*	31	14.33 ± 0.57	0	0	12.66 ± 1.15	0	0
*S. flexenari*	33	0	0	0	11.33 ± 1.15	0	0
*S. aureus*	44	14.33 ± 0.57	0	0	22.33 ± 0.57	0	0
*P. auriginosa*	26	14.33 ± 0.57	0	0	0	0	0
*S. typhi*	41	0	0	0	0	0	0

6–9 mm: no activity; 12–15 mm: low activity; 15–18 mm: good activity; above 18 mm: significant activity.

**Table 2:** Antibacterial activity of crude extract and fractions of leaves (Imipenem as standard drug).

Bacteria	Standard drug zone of inhibition	Crude extract zone (mm)	*n*-hexane zone (mm)	Chloroform zone (mm)	Ethyl acetate zone (mm)	*n*-butanol zone (mm)	Aqueous zone (mm)
*E. coli*	30	0	0	0	0	0	0
*B. subtillis*	31	8.66 ± 0.57	0	0	0	0	0
*S. flexenari*	33	0	0	0	7.33 ± 0.57	0	0
*S. aureus*	44	0	0	0	9.33 ± 1.54	0	0
*P. auriginosa*	26	7.66 ± 0.57	0	0	0	0	0
*S. typhi*	41	0	0	0	0	0	0

6–9 mm: no activity; 12–15 mm: low activity; 15–18 mm: good activity; above 18 mm: significant activity.

**Table 3:** Antifungal activity of crude extract and fractions of rhizomes (Miconazole as standard drug).

Fungi	−ve control	Standard MIC (*μ*g/mL)	Crude extract LG (mm)	*n*-hexane LG (mm)	Chloroform LG (mm)	Ethyl acetate LG (mm)	*n*-butanol LG (mm)	Aqueous LG (mm)
*T. longifusus*	100	70	100	100	100	100	100	100
*C. albicans*	100	110.8	40	100	100	50	100	100
*M. canis*	100	98.4	35	100	100	48	100	20
*A. flavusi*	100	20	100	100	100	100	100	100
*F. solani*	100	73.3	25	100	100	30	100	100

Inhibition of 0–40%: no activity; 40–60%: low activity; 60–70%: moderate activity; 70–100%: significant activity; LG: linear growth (mm).

**Table 4:** Cytotoxic activity of *G. wallichianum* rhizome crude extract and fractions.

Sample	No. of shrimps	Number of lethality			LC_50_
		1000 **μ**g/mL	100 **μ**g/mL	10 **μ**g/mL
Crude extract	30	3	0	0	>1000
Chloroform	30	0	0	0	>1000
Ethyl acetate	30	25	3	1	333.256
*n*-butanol	30	0	0	0	>1000
Aqueous	30	2	1	0	>1000

**Table 5:** Phytotoxic activity of *G. wallichianum* rhizome crude extract and fractions.

Sample	Concentration (**μ**g/mL)	Number of fronds survived		% Growth regulation	Standard paraquat
		Sample	Control		
Crude extract	10	0	25	100	100
	100	0	23	100	100
	1000	0	26	100	100
Chloroform fraction	10	20	25	20	100
	100	18	23	21.73	100
	1000	15	25	40	100
Ethyl acetate fraction	10	20	25	20	100
	100	20	23	13.04	100
	1000	19	26	26.92	100
*n*-butanol fraction	10	20	25	20	100
	100	19	23	17.39	100
	1000	19	26	26.92	100
Aqueous fraction	10	19	25	24	100
	100	19	23	17.39	100
	1000	18	25	28	100

Inhibition of 30–40%: nonsignificant activity; 50%: moderate activity; 60–70%: good activity; 70%: significant activity.

**Table 6:** Insecticidal activity of *Geranium wallichianum* rhizome crude extract and fractions.

Sample	Number of lethality (%)					
	*Tribolium castaneum*		*Rhyzopertha dominica*		*Callosbruchus analis*	
Crude extract	0.33 ± 0.57	3.3 ± 5.7	0	0	0	0
Chloroform	1.33 ± 0.57	13.3 ± 5.7	4.66 ± 0.57	46.6 ± 5.7	0	0
Ethyl acetate	0.66 ± 0.57	6.6 ± 5.7	6.66 ± 1.15	66.6 ± 11.5	6.6 ± 5.7	66.6 ± 0
*n*-butanol	0	0	0	0	0.66 ± 0.57	6.6 ± 5.7
Aqueous	0	0	1.33 ± 0.57	13.3 ± 5.7	0	0
Permethrin (standard)	10 ± 0	100	10 ± 0	100	10 ± 0.01	100
Saline	0	0	0	0	0	0

No. of observations (*n*) is 10 in each case.

**Table 7:** Enzyme inhibition studies on rhizome extract and fractions of *G. wallichianum*.

Enzyme	Inhibition (%)				
	Crude extract	Chloroform	Ethyl acetate	*n*-butanol	Aqueous
Lipoxygenase	26.9	0	47.5	0	45
J.B. urease	52	0	83	0	0
B.P. urease	57	0	86	0	0

**Table 8:** Enzyme inhibition studies on pure compounds **1**–**6** from *G. wallichianum*.

Compounds	Inhibition (%)		
	Lipoxygenase	J.B. urease	B.P. urease
Ursolic acid (**1**)	0	52	57
**β**-sitosterol (**2**)	35	0	0
Stigmasterol (**4**)	37.5	0	0
**β*-*sitosterol glactoside (**3**)	40	0	0
2,4,6-trihydroxyethylbenzoate (**6**)	100	66	69
Herniarin (**5**)	0	0	0
